# Supplementary material for: Ecological drivers of African swine fever virus persistence in wild boar populations: Insight for control
Source: Ecol Evol. 2020 Feb 18;10(6):2846–59. doi: 10.1002/ece3.6100 (PMC7083705; doi:10.1002/ece3.6100)
Supplement: Supplementary file 1 [file ECE3-10-2846-s001.pdf]

Ecological drivers of African swine fever virus persistence in wild boar populations: insight for control.

Kim M. Pepin[1,*], Andrew J. Golnar[1], Zaid Abdo[2] and Tomasz Podgórski[3,4]

Supplementary Figures

Fig. S1. Schematic of modeling approach.

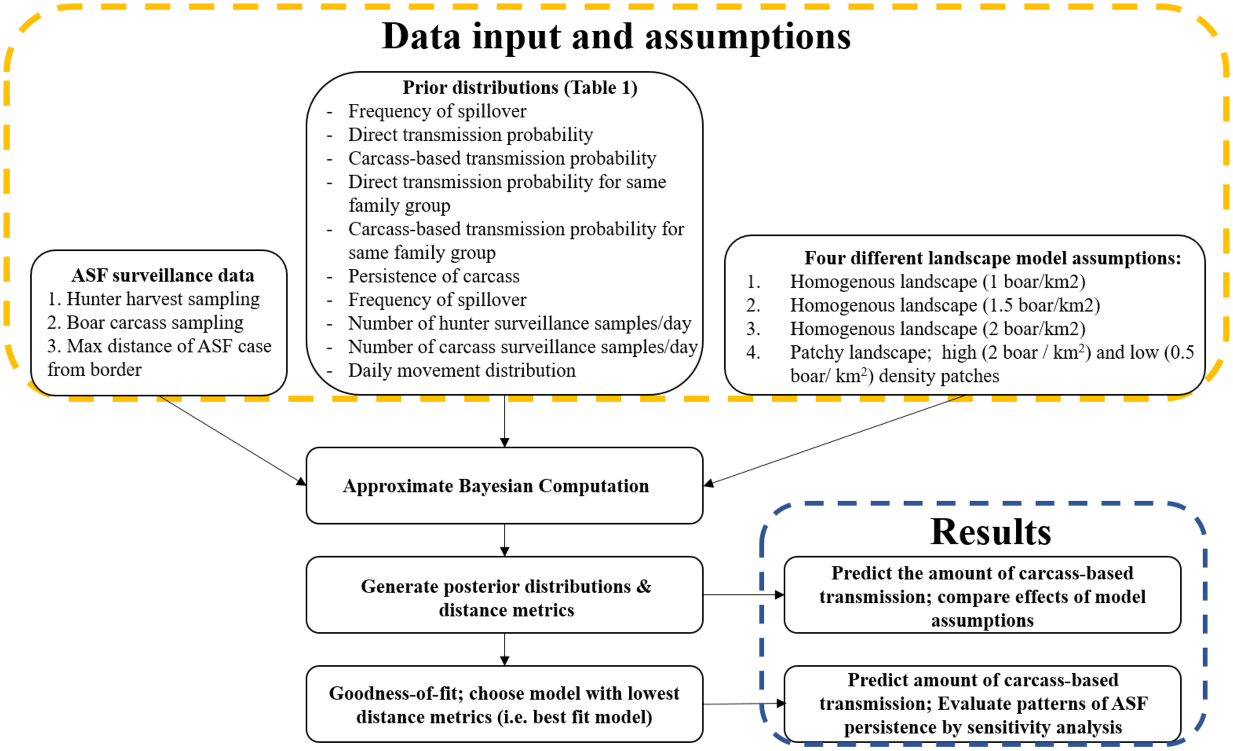

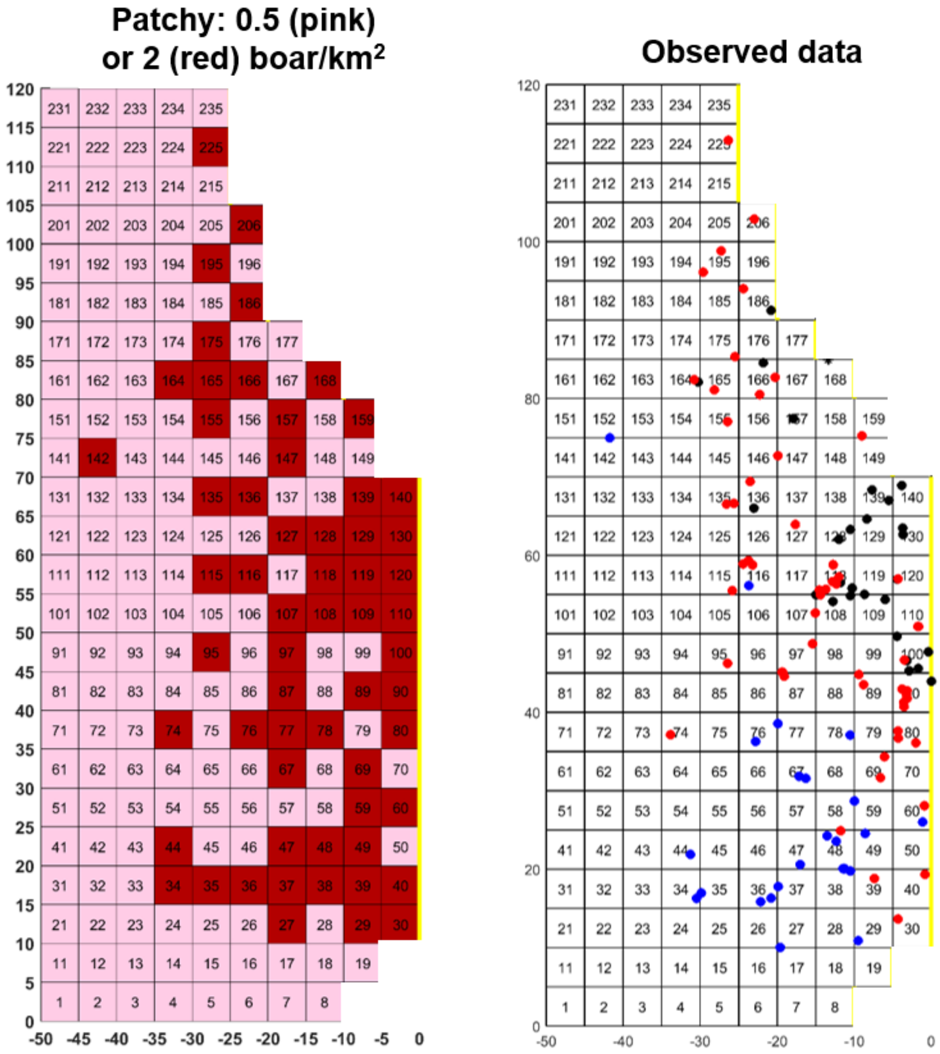

Fig. S2. Host density structure of landscapes used for data fitting by ABC. X and Y-axes are in km. The Eastern border is indicated in yellow – boar were not allowed to the right of this line. Each grid cell is 5 x 5 km. Grid cell colors correspond to maximum boar density allowed in the grid cell which was 0.5 (light pink) or 2 (dark red) boar / km^2. This landscape was the patchy landscape. We also compared fits on this landscape to ones with homogenous densities of 1 (the average of the patchy landscape), 1.5, and 2 boar/km^2 to evaluate the role of landscape structure in explaining the patterns of spread. The grid on the right shows observed case locations by year:

2014 (black), 2015 (red), Jan.-Jul. 2016 (blue). The data from 2016 was withheld for parameter estimation, but was used to assess out-of-sample model performance.

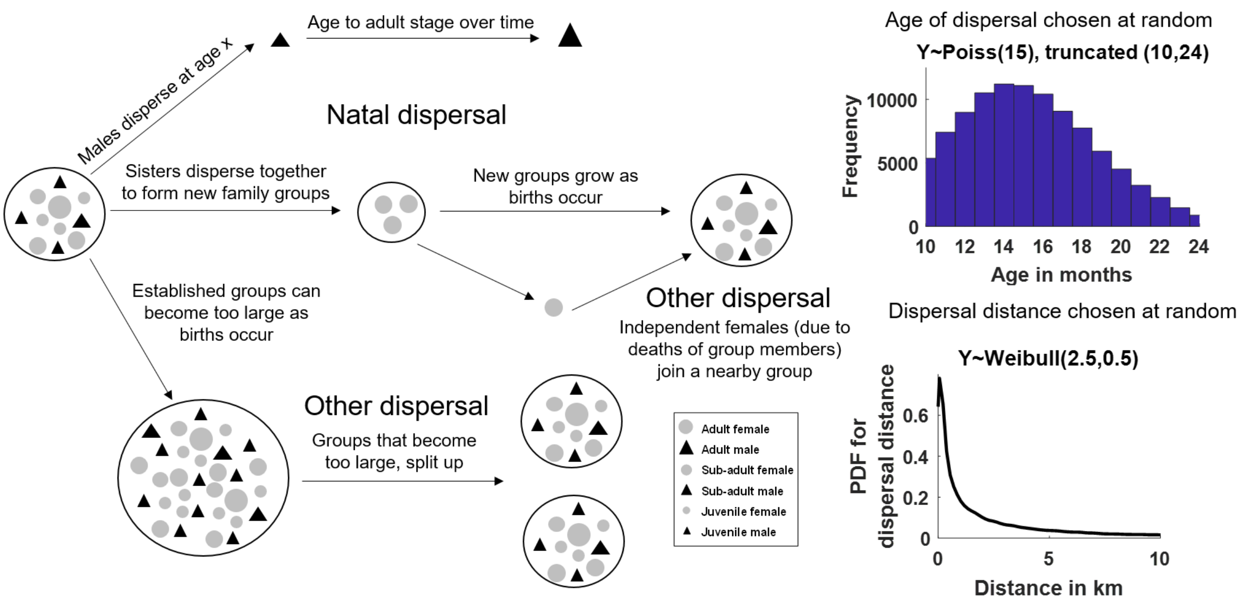

S3. Dispersal and social dynamics. Schematic of dispersal mechanisms and social dynamics. These dispersal events result in permanent relocation of the home range centroid. Natal dispersal age and dispersal distance for each permanent relocation event are chosen at random from distributions supported by empirical data displayed on the right half of the figure.

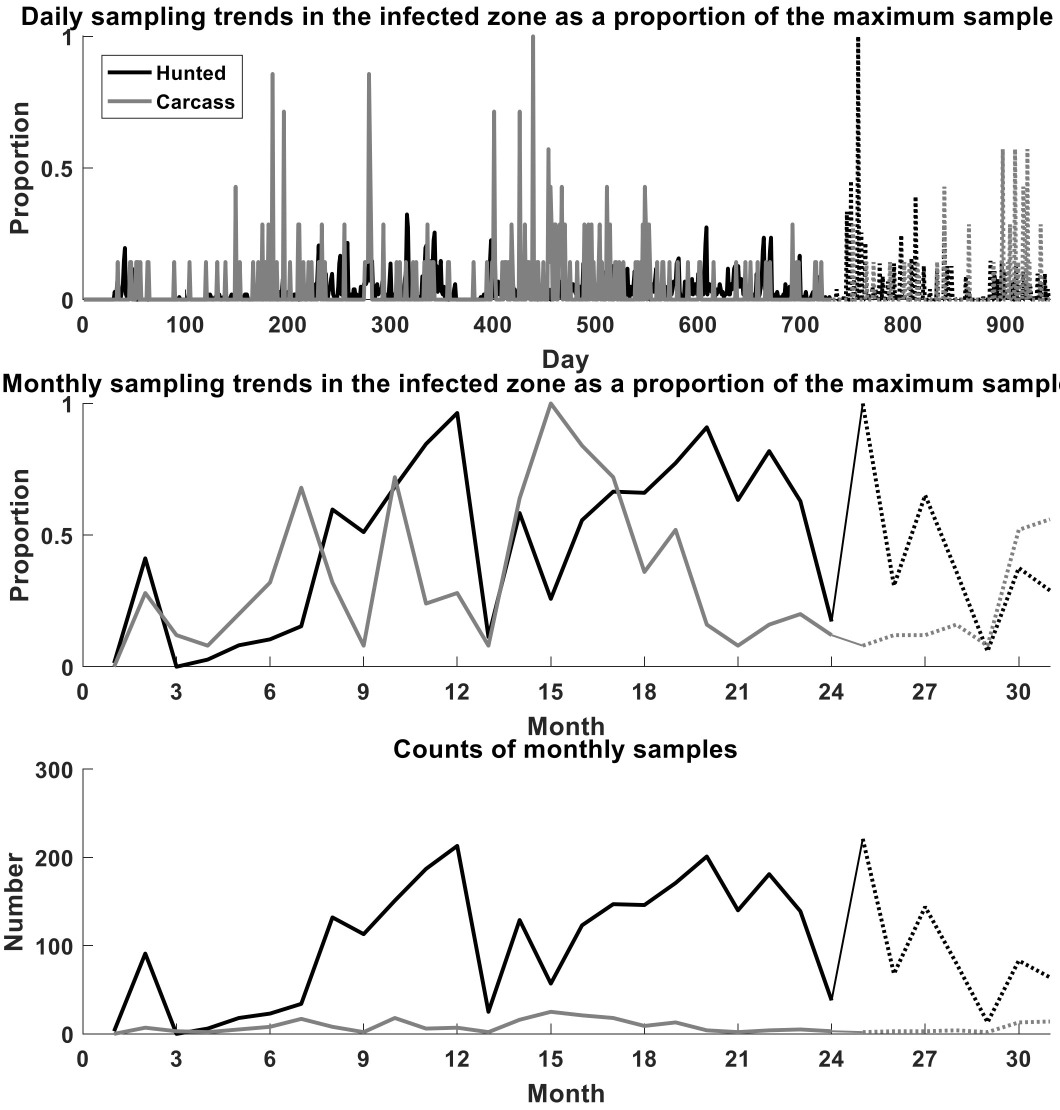

Fig. S4. Sampling trends in the infected zone. The vectors of daily proportion values were multiplied by scaling parameters (ρ_h, ρ_c) to determine the proportion of the population sampled each day. Thus, for example, the number of boar sampled on day *t* by hunting was: number of boar on the landscape (only including those > 6 months) x proportion in the sampling trend vector on day *t* x ρ_h. This method accounts for seasonal changes in boar abundance and sampling. The dotted lines indicate 2016 data, which were not used for parameter estimation but were used for prediction out-of-sample.

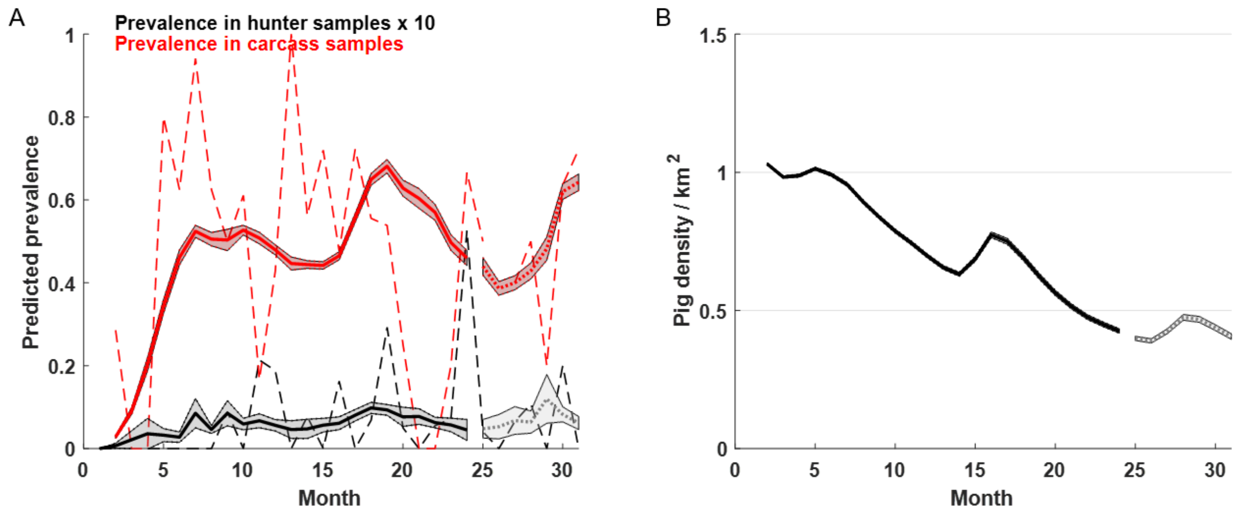

Fig. S5. Predictions from 1000 simulations from the posterior distribution (patchy landscape). Solid lines indicate the time period used for model fitting whereas dotted lines show the out-of-sample predictions for 2016. A. Monthly prevalence in hunted (black) and carcass (red) surveillance samples. Dashed lines are monthly prevalence in the real surveillance data. B. Predicted abundance of wild boar in the simulations.

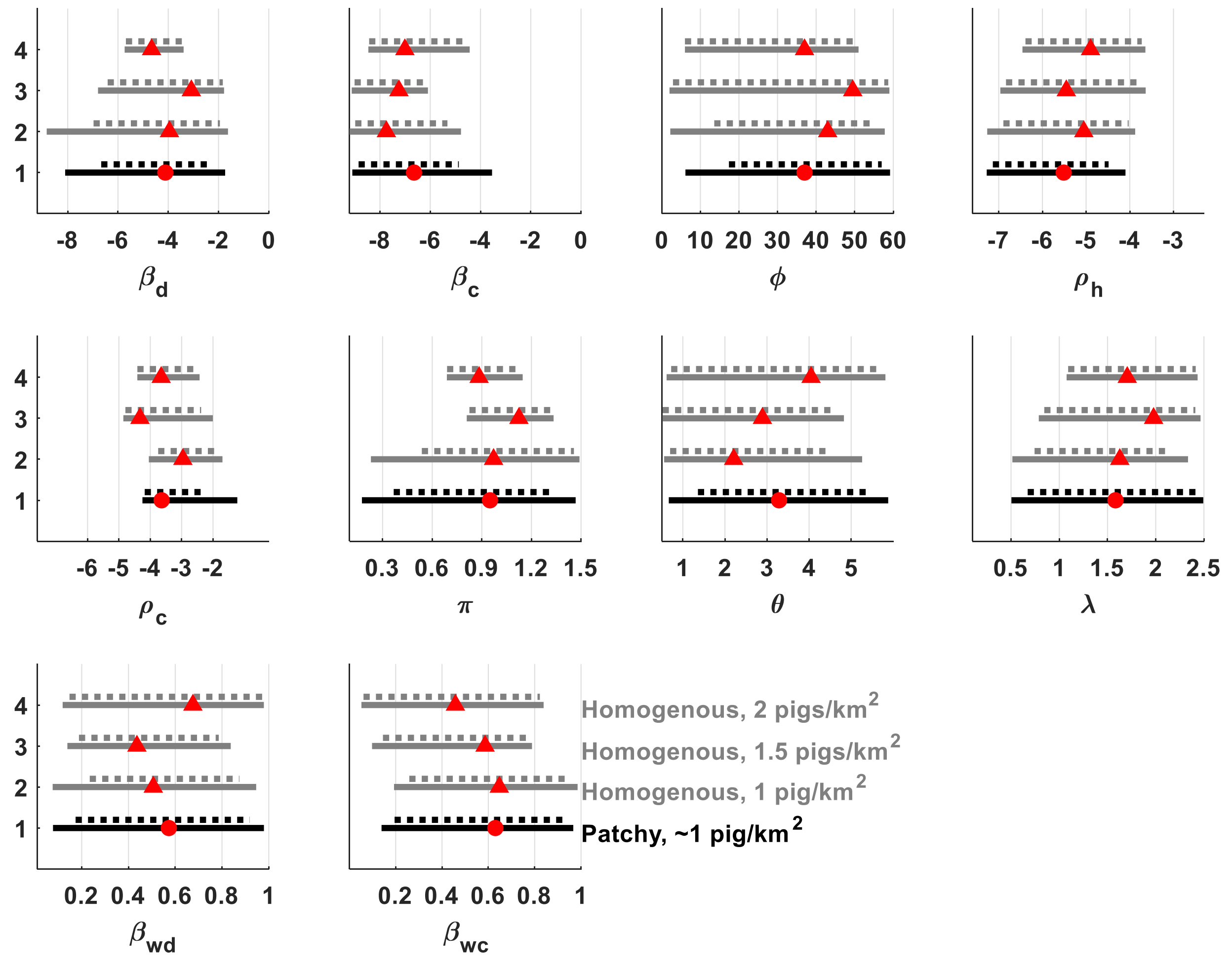

Fig. S6. Credible intervals (CI) of estimated parameters for each model (solid lines are 95% CIs, dotted lines are 80% CIs). Prior distributions are all uniform and span the width of the X-axes. Red shapes indicate the median of the posterior distribution.

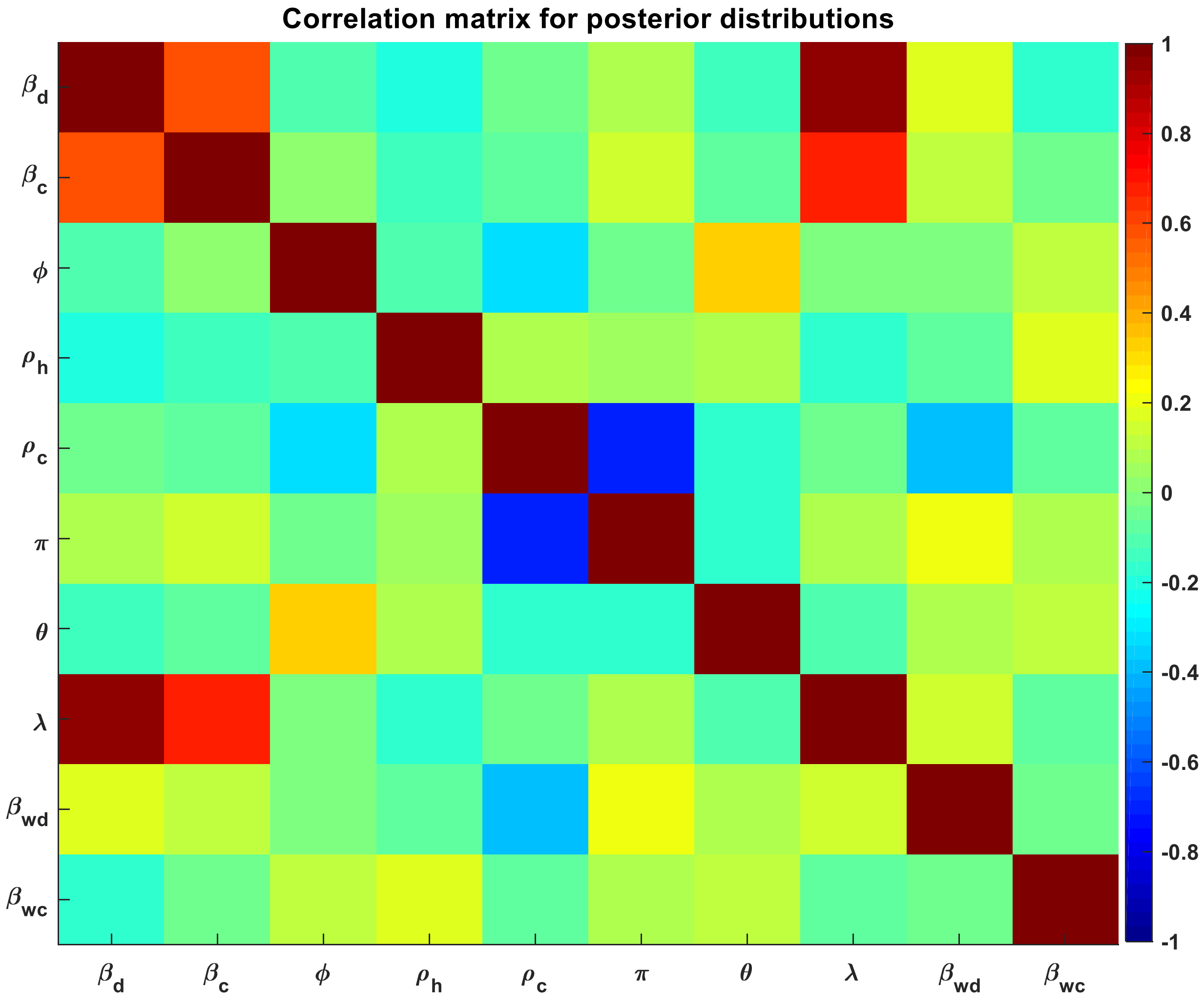

Fig. S7. Results are from the patchy landscape. Colors are correlation coefficients between parameters. β_d and β_c were positively correlated with each other and even more positively correlated with λ, whereas ρ_c and π were negatively correlated, and other parameters were relatively uncorrelated.
