# Supplementary material for: Ecological drivers of African swine fever virus persistence in wild boar populations: Insight for control
Source: Ecol Evol. 2020 Feb 18;10(6):2846–59. doi: 10.1002/ece3.6100 (PMC7083705; doi:10.1002/ece3.6100)
Supplement: Supplementary file 2 [file ECE3-10-2846-s002.pdf]

```
function [update, del] = density_dep_disp_v4(X,id,dd,net_list) %,moredata: an additional outp
ut for tracking dispersal distance
% This function operates per dispersal event (i.e., for one group or
% individual going to the same place)
del = [];

        ang = degtorad(0:45:315);
        % get population size in each cell
        pops = histc(X(:,27),net_list(:,1));
        getinfo = zeros(length(ang),size(net_list,2)+1); % add a clolumn to label whether thi
s is a valid destination
        getinfo(:,1) = 1;
        crit = -1;
        dist = dd/2;
        while crit <= 0 && min(getinfo(:,1)) > 0 % if all candidates can't accept the group a
nd none are off the grid, go twice as far
            dist = dist*2;

            if dist > 10
                getinfo = [zeros(size(getinfo,1),size(net_list,2)) ones(size(getinfo,1),1)];
                break
            end
                    x = dist.*cos(ang)+X(id(1),17); % get list of potential points for disper
sal
                    y = dist.*sin(ang)+X(id(1),18);
                    %%%%%%%%%%%%%%%%%%%
                for jj = 1:length(ang) % Determine grid cell id for each potential point
                    id_cell = find(net_list(:,4) < x(jj) & net_list(:,6) > x(jj) & net_list(:
,5) < y(jj) & net_list(:,7) > y(jj));
                    if isempty(id_cell) == 0
                        getinfo(jj,1:size(net_list,2)) = net_list(id_cell,:); %only store inf
o if it's on the grid
                        ind = find(net_list(:,1) == getinfo(jj,1)); % get id for this grid ce
ll
                        getinfo(jj,size(getinfo,2)) = (getinfo(jj,8)-(pops(ind)+length(id)))
>= 0;
                    else
                        getinfo(jj,:) = [0 0 0.5 zeros(1,size(net_list,2)-3) 1]; % for off-th
e-grid locations, set probability to 0.5
                    end

                end
                % reset distances to go twice as far if needed
                    crit = max(getinfo(:,size(getinfo,2))); % for getinfo(:,1) > 0, find pop
size + new potentials that can fit in new location
        end

% Choose the valid destination for dispersal
            celltogoids = find(getinfo(:,size(getinfo,2)) > 0); % get all valid moves
            if length(celltogoids) == 1
                celltogo = celltogoids;
            else
                celltogo = randsample(celltogoids,1); % choose one at random (weight by habit
at for habitat dependent models)
            end
```

```
                 if getinfo(celltogo,1) == 0
                    del = [del id]; update = nan;
                 else % need to extract actual distances moved
                        x2 = rand*(getinfo(celltogo,6)-getinfo(celltogo,4)) + getinfo(celltog
o,4);
                        y2 = rand*(getinfo(celltogo,7)-getinfo(celltogo,5)) + getinfo(celltog
o,5);
                        update = [x2 y2 getinfo(celltogo,[1 8])];  % new home range, habitat
id and cell K
                        del = [];
                 end

end
```

```
Not enough input arguments.

Error in density_dep_disp_v4 (line 8)
        pops = histc(X(:,27),net_list(:,1));
```
