# Supplementary material for: Ecological drivers of African swine fever virus persistence in wild boar populations: Insight for control
Source: Ecol Evol. 2020 Feb 18;10(6):2846–59. doi: 10.1002/ece3.6100 (PMC7083705; doi:10.1002/ece3.6100)
Supplement: Supplementary file 3 [file ECE3-10-2846-s003.pdf]

Contents

- DISEASE TRANSMISSION
- NATURAL MORTALITY %%
- DISPERSAL AND SOCIAL DYNAMICS %%
- SAMPLING
- NEW CONCEPTIONS - Density-dependent conception %%
- NEW BIRTHS %%%
- SUMMARIZE THE OUTPUT


```
%Main function for simulating host populaiton and disease dynamics
%%%%%%%%%%%%%%%%%%%%%%%%%%%%%%%%%%%%%%%%%%%%%%%%%%%%%%%%%%%%%%%%%%%%%%%
%MATRIX FOR TRACKING INDIVIDUAL STATUS
% pig ID = 1
% Age = 2
% Sex = 3
% Group ID = 4
% Dispersal status = 5 (0 = not yet, 1 = already)
% Dispersal age = 6
% [OPEN] = 7
% Age at natural death = 8
% Age at first conception = 9
% Litter size = 10
% Gestation period = 11
% Reproductive status = 12
% Pregnancy clock = 13
% Post natal clock = 14
% [OPEN] = 15
% [OPEN] = 16
% Home range x = 17
% Home range y = 18
% [OPEN] = 19
% Total litters so far = 20
% Total offspring so far = 21
% Sterilization Status = 22
% S = 23 (susceptibility status)
% E = 24 (clock for E)
% I = 25 (clock for I)
% R = 26 (clock for C)
% Spatial grid ID = 27
% Cell-specific total K = 28
% Clock for uninfected Carcass = 29

%SAMPLING:
% Rescale daily sample sizes so they are a proportion of the maximum value (this gives variat
ion in sampling intensity)
% Multiply the sampling intensity by the total population size and a detection probability to
 determine the actual sample size to be collected
% Do this for live and dead individuals separately
% Sample individuals at random in space
% Record the nummber that are positive and negative.
```

```
%%%%%%%%%%%%%%%%%%%%%%%%%%%%%%%%%%%%%%%%%%%%%%%%%%%%%%%%%%%%%%%%%%%%%%%%%%%
% OUTPUTS:
%store(time,29): for each time step
% 1:8:  0-1, 1-2, 2-3, 3+ females, 0-1, 1-2, 2-3, 3+ males,
% 9:10:  number of sounders, average size of sounders,
% 11:13:  number of solo males, number new conceptions, number of new births,
% 14:15:  number of new solo males, new sounders,
% 16:  number of groups of young males;
% 17:18: number and proportion of natural deaths
% 19:20: number and proportion of culled individuals
% 21:22: number and proportion of newly sterilized individuals
% 23:24: group members that move due to overcrowding, solomales that move due to overcrowding
% 25: habitat-dependent mortality
% 26: number leaving grid
% 27: number dead due to disease
% 28:29: maximum transmission distance allowed at each time step for direct and carcass
%

function [met,timeseries,FOI,deg,SEIR,Re] = main_predict_DenDepRepro2(time,X,D_age,D_d,L_size
,L_age,min_P,gest,long,...
    c_time,tinfect,net_list,alpha,gamma,zeta,sz,TPGC_d,TPGC_c,K,samples,dp,truth,...
    bordercoor,movement,parmsin,ndeg) %int_cull,prop_cull,space_cull,num_cull,int_vac,prop_va
c,space_vac,num_vac,

% popthreshold = size(X,1)*0.2; %set an abundance threshold that when pop goes below this sto
p (10 % of initial pop size)
% Ecut = 150; %if there are more new cases than this per day than stop
% Ocut = 300; % if the total observed cases go beyond this than stop

ss = [samples(:,4) sum(samples(:,5:6),2)]; %inf zone
sampprob = zeros(size(ss,1),2);
sampprob(:,1) = (ss(:,1)./max(ss(:,1))).*dp(1);
sampprob(:,2) = (ss(:,2)./max(ss(:,2))).*dp(2);

zeta = zeta.*sz;
%%%%%%%%%%%%%%%%%%%%%%%%%%%%%%%%%%%%%%%%%%%%%%%%%%%%%%%%%%%%%%%%%%%%%%%%%%%%%%%%%%%%%%%%%%%%%
%%%%%%%%%%%%%%%%%%%%%%%%%%%%%%%%%%%%%%%%%%
%%%%%%%%%%%%%%%%%%%%%%%%%%%%% START THE MAIN SIMULATION LOOP %%%%%%%%%%%%%%%%%%%%%%%%%%%%%%%%
%%%%%%%%%%%%%%%%%%%%%%%%%%%%%%%%%%%%%%%%%%
%%%%%%%%%%%%%%%%%%%%%%%%%%%%% %%%%%%%%%%%%%%%%%%%%%%%%%%%%%%%%%%%%%%%%%%%%%%%%%%%%%%%%%%%%%%%
%%%%%%%%%%%%%%%%%%%%%%%%%%%%%%%%%%%%%%%%%%
%StoreGrid = zeros(size(net_list,1),time,3); % 1: abundance by grid cell; 2: new cases by gri
d cell, 3: infectious individuals by grid cell
SEIR = zeros(time,5);
FOI = zeros(time,6);
samp = zeros(time,2); % store sample sizes collected
death = zeros(time,4); %natural, density-dep, disease, sampling
timeseries = zeros(time,4); %cases, abundance, proptrans
indexcases = []; %R0 = 0; %maxuniqueid = max(X(:,1));
surv = nan(time,8);%live, dead pos, neg, distance
%abundance = nan(time,4);
%data = zeros(50000,9);
intros = 1;
%TIME FRAME FOR AGGREGATION
enddate = [31 28 31 30 31 30 31 31 30 31 30 31 31 28 31 30 31 30 31 31 30 31 30 31 31 29 31 3
0 31 30 31]; %month ends to end of July 2016
```

```
cumdate = cumsum(enddate);
checkvec = ismember(1:time,cumdate);
check1 = []; % positive samples live
endi = time+1;
cnet = zeros(500,time,2); % recipient and donor ids each day
uindID = [unique(X(:,1)) nan(length(unique(X(:,1))),1)]; %list of all unique ids that existed
 at some point (this will make up the transmission network matrix)

for i = 1:time % For each day
    % update unique list of individuals
    uid = unique(X(:,1)); match = find(ismember(uid,uindID(:,1))==0);
    uindID = [uindID; [uid(match) nan(length(match),1)]];
    if min(endi) > time
        %abundance(i,:) = [length(find(X(:,26) == 0 & X(:,29) == 0)) length(find(X(:,26) > 0
& X(:,29) == 0)) length(find(X(:,26) == 0 & X(:,29) > 0)) length(find(X(:,26) > 0 & X(:,29) >
 0))];
        %StoreGrid(:,i,1) = hist(X(:,27),net_list(:,1))'; % Store nummber of pigs in each cel
l
        %StoreGrid(:,i,3) = hist(X(X(:,25)>0 | X(:,26)>0,27),net_list(:,1))'; % Store nummber
 of infectious pigs in each cell

    %sounders = length(unique(X(X(:,4)>0,4))); % current number of family groups
    X(:,2) = X(:,2)+1; % update ages
    id = find(X(:,13) > 0); X(id,13) = X(id,13)+1; %update pregnancies
    id = find(X(:,3) == 0 & X(:,9) < X(:,2) & X(:,12) == 0 & X(:,22) == 0); X(id,12) = 1; % u
pdate reproductive status of non-sterilized females
    id = find(X(:,14) > 0); X(id,14) = X(id,14)+1; %update postnatal clock for non-pregnants

   %%%%%%%%%%%%%%%%%%%%%%%%%%%%%%%%%%%%%%%%%%%%%%%%%%%%%%%%%%%%%%%%%%%%%%%%%%%
```

DISEASE TRANSMISSION

```
            if (sum(X(:,25)) > 0 || sum(X(:,26)) > 0) && sum(X(:,23)) > 0
            [DATA,Sid,Eid,Eidc,pairs] = dis3(X,TPGC_d,TPGC_c,movement,i);
                % update new exposures
                SEIR(i,5) = length(Eid)+length(Eidc); %record new exposures
                X(Sid(Eid),23) = 0; % S to E
                X(Sid(Eid),24) = max(1,poissrnd(alpha,length(Sid(Eid)),1)); % Poisson-distrib
uted incubation period
                X(Sid(Eidc),23) = 0; % S to E
                X(Sid(Eidc),24) = max(1,poissrnd(alpha,length(Sid(Eidc)),1)); % Poisson-distr
ibuted incubation period
                % Calculate FOI (whole landscape vs only cells with at least one E/I/C indivi
dual)
                LiveI = length(find(X(:,25)>0));
                DeadI = length(find(X(:,26)>0));
                %disp([LiveI DeadI])
                infcellsL = X(find(X(:,25)>0),27); % Get all individuals from cells that have
 E I or C
                infcellsC = X(find(X(:,26)>0),27); % Get all individuals from cells that have
 E I or C
                Sreduced = ismember(X(Sid,27),[infcellsL;infcellsC]);
                FOI(i,:) = [(LiveI+DeadI)/length(Sid) (LiveI+DeadI)/sum(Sreduced) LiveI/lengt
h(Sid) DeadI/length(Sid) LiveI/sum(Sreduced) DeadI/sum(Sreduced)];
                %R0 = R0+R0tally; %update R0
                % Record location of new cases
```

```
                if nansum(nansum(DATA)) > 0
%                     id = find(data(:,1)==0,1,'first');
%                     data(id:id+size(DATA,1)-1,:) = DATA;
                    numdir = length(find(DATA(:,9) == 1));
                    numcarc = length(find(DATA(:,9) == 2));
                    proptrans = numdir./(numdir+numcarc);
                    temp = DATA(:,6:7);
                    mat = sqrt((temp(:,1)-repmat(bordercoor(:,1)',size(temp,1),1)).^2 + (tem
p(:,2)-repmat(bordercoor(:,2)',size(temp,1),1)).^2);
                    dd = min(mat,[],2); % true distance from the border for each case
                   timeseries(i,:) = [SEIR(i,5) length(find(X(:,26) == 0 & X(:,29) == 0)) pr
optrans max(dd)];
                else
                    timeseries(i,:) = [0 length(find(X(:,26) == 0 & X(:,29) == 0)) NaN NaN];
                end

                newc = [X(Eid,1);X(Eidc,1)]; % get ids of new cases so we can track the Re fo
r infectious individuals
                match = find(ismember(uindID(:,1),newc)==1); %find the new cases in the full
list of unique IDs and set their transmission counts to 0
                uindID(match,2) = 0;

                if isempty(pairs) < 1
                    cnet(1:size(pairs,1),i,1) = pairs(:,1); %recipient, donor
                    cnet(1:size(pairs,1),i,2) = pairs(:,2);
                    upairs = unique(pairs(:,2));
                    for iii = 1:length(upairs)
                        id = find(uindID == upairs(iii));
                        uindID(id,2) = uindID(id,2)+sum(pairs(:,2) == upairs(iii)); % add a t
ransmission event for each S that each I transmits to
                    end
                end


                clear DATA Sid Eid Eidc;

            end

            if sum(sum(X(:,24:26))) > 0 %if there are any disease clocks to deal with

            % make transitions E-I, I-C, C-D & update clocks: work backwards
            Rc = find(X(:,26) > 1); % id for those with a clock not about to transition to de
ad
            Rt = find(X(:,26) == 1); % id for those who will transition from carcass to dead
            Ic = find(X(:,25) > 1);  % id for those with a clock not about to transition from
 I to carcass
            It = find(X(:,25) == 1);  % id for those who will transition from I to carcass
            Ec = find(X(:,24) > 1);  % id for those with a clock not about to transition
            Et = find(X(:,24) == 1);  % id for those who will transition from E to I

            X(Et,24) = 0; % E to I
            X(Et,25) = max(1,poissrnd(gamma,length(Et),1));
            X(It,25) = 0; % I to C
            death(i,3) = length(It); % death due to disease
            X(It,26) = max(1,poissrnd(zeta(i),length(It),1));

            % update clocks
```

```
            X(Rc,26) = X(Rc,26)-1; % CARCASS clock
            X(Ic,25) = X(Ic,25)-1; % I clock
            X(Ec,24) = X(Ec,24)-1; % E clock
            X(Rt,:) = []; % Kill the carcasses that are done being infectious
            end

    %%%%% INITIATE DISEASE (and migration of disease)
     if tinfect(i) > 0 % introduce an infectious individual
         Z = zeros(1,size(X,2));
            Z([27 28]) = [tinfect(i) net_list(tinfect(i),8)]; % assign grid cell where they w
ill appear
            Z(24) = max(1,poissrnd(alpha,1)); % set alpha to be at least one day
            Z(1) = max(X(:,1))+1; % assign unique id
            Z(2) = randsample(long,1); Z(1,8) = randsample(long,1)+365; % assign age and make
 sure they live long enough to transmit
            Z(3) = randsample([0 1],1); % assign sex
            Z(6) = randsample(D_age,1); if Z(2) > Z(6); Z(5) = 1; else; Z(5) = 0; end; %set d
ispersal age/status
            if Z(2) == 1 && Z(3) == 1; Z(4) = NaN; else; Z(4) = max(X(:,4))+1; end; % assign
group id
            if Z(3) == 0; Z([9 14]) = [L_age min_P]; end; % if it's a female, assign the repr
oductive info
            HRx = rand*(net_list(tinfect(i),6)-net_list(tinfect(i),4)) + net_list(tinfect(i),
4); % get x coordinate for home range centroid of pigs
            HRy = rand*(net_list(tinfect(i),7)-net_list(tinfect(i),5)) + net_list(tinfect(i),
5);
            Z(15:18) = [HRx HRy HRx HRy]; % give it some coordinates
            X = [X;Z]; % add it to the population
            intros = intros-1; %Mark the intro sequence (index case will be 0)
%             iddata = find(data(:,1)==0,1,'first');
%             data(iddata,:) = [i Z([1 2:4 17 18 27]) intros]; % record data
            timeseries(i,:) = [1 length(find(X(:,26) == 0 & X(:,29) == 0)) -1 0];
            uindID(uindID==Z(1),2) = 0; % set it's transmission events to 0 so we can start c
ounting them
     end
```

NATURAL MORTALITY %%%

```
    id = find(X(:,2) > X(:,8) & X(:,26) == 0 & X(:,29) == 0); % get the ones that reach their
 age of natural death (don't include infectious carcasses)
    death(i,1) = length(id); %store number of natural deaths
    X(id,[4 29]) = [nan(length(id),1) max(1,poissrnd(zeta(i),length(id),1))]; % dissociate fr
om group, start carcass clock (amount of time carcass is available for detection
    id = find(X(:,29)>0); X(id,29) = X(id,29)-1; % tick the carcass clock
    X(X(:,29)==1,:) = []; % erase the ones that have been on the landscape too long
```

DISPERSAL AND SOCIAL DYNAMICS %%%

MALE DISPERSAL - ALL DISPERSE BY THEMSELVES

```
    mids = find(X(:,2) > X(:,6) & X(:,5) == 0 & X(:,26) == 0 & X(:,29) == 0 & X(:,3) == 1); %
 get male dispersers
    if isempty(mids) == 0 % all males disperse by themselves
```

```
        offgrid = [];
        for ww = 1:length(mids)
        [update, del] = density_dep_disp_v4(X,mids(ww),randsample(D_d,1),net_list);
          if isempty(del) == 1 % if they are staying on the grid, update info
            X(mids(ww),4) = nan; % for males, go on their own
            X(mids(ww),[5 17 18 27 28]) = [1 update]; % change dispersal status to done, upda
te attributes
          else % otherwise, mark them for deletion
              offgrid = [offgrid; mids(ww)];
          end
        end
        X(offgrid,:) = []; % delete the ones going off the grid
    end % if isempty(ids)

% FEMALE DISPERSAL - ALL DISPERSE IN GROUPS
    fids = find(X(:,2) > X(:,6) & X(:,5) == 0 & X(:,26) == 0 & X(:,29) == 0 & X(:,3) == 0); %
 get female dispersers
    mat = [fids X(fids,4)]; % link to same group

    if isempty(fids) == 0
        ugrp = unique(mat(:,2));
        offgrid = [];
        for ww = 1:length(ugrp)
            tempid = mat(mat(:,2) == ugrp(ww),1); % set of ids for group ww
            if length(tempid) > 2 % only disperse the females if they are more than 2 going t
ogether
                [update, del] = density_dep_disp_v4(X,tempid(1),randsample(D_d,1),net_list);
                if isempty(del) == 1 % if they are staying on the grid, update info
                    X(tempid,4) = max(X(:,4))+1; % for females, make a new sounder
                    X(tempid,[5 17 18 27 28]) = repmat([1 update],length(tempid),1); % change
 dispersal status to done, update attributes
                else % otherwise, mark them for deletion
                    offgrid = [offgrid; tempid];
                end
            else % otherwise just reset their dispersal to done and keep the rest as is
                X(tempid,5) = 1;
            end
        end
        X(offgrid,:) = []; % delete the ones going off the grid
    end

% SOCIAL DYNAMICS: RELOCATION DUE TO OVERSIZED FAMILY GROUPS (SPLIT SOUNDERS IN HALF, AT
% LEAST 1 MATURE FEMALE PER GROUP LEAVES WITH YOUNGER ONES)
    temp = X(X(:,26)==0 & X(:,29) == 0 & X(:,4) > 0,:);
    ufem = [histc(temp(:,4),unique(temp(:,4))) unique(temp(:,4))]; %# of ALIVE individuals in
 each family group
    g_id = ufem(ufem(:,1) > K(1),2); %get family group id's that have grown > K
    if isempty(g_id) == 0 % if there are some
        offgrid = [];
        for k = 1:length(g_id) % for each family group that got too big, disperse a proportio
n of the group to form a new group
            % by choosing a random individuals
            all = find(X(:,4) == g_id(k)); % get all members in the group
            temp = sortrows([all X(all,2)],2); % sort so youngest are first (choose youngest
to leave with an adult)
            id_move = [temp(1:round(K(1)/2)-1,1); temp(size(temp,1),1)]; % choose half of the
 group to relocate (the oldest plus a group of the youngest)
```

```
            if isempty(id_move) == 0 % assign the group a new location
                [update, del] = density_dep_disp_v4(X,id_move(1),randsample(D_d,1),net_list);
                    if isempty(del) == 1 % if they are staying on the grid, update info
                        X(id_move,[4 5 17 18 27 28]) = [(max(X(:,4))+1).*ones(length(id_move)
,1) ones(length(id_move),1) repmat(update,length(id_move),1)]; % change dispersal status to d
one for females, update attributes
                        X(X(id_move,3)==1 & X(id_move,5)==0,5) = 0; % makeit so non-dispersed
 males can still disperse to become independent
                    else % otherwise, mark them for deletion
                        offgrid = [offgrid; id_move];
                    end
            end
        end
        X(offgrid,:) = []; % delete the ones going off the grid
    end

%%%%%%%%%% CHECK THAT NO FEMALES ARE IN A GROUP ALONE - ASSOCIATE TO NEAREST
    % GROUP
    [~, ~, xc] = intersect(unique(X(X(:,4)>0,4)),X(:,4));
    if length(xc) > 1 %size(hist(X(X(:,4)>0,4),unique(X(X(:,4)>0,4)))',1) == size(unique(X(X(
:,4)>0,4)),1) %DEBUGGING CHECK
    group = [hist(X(X(:,4)>0,4),unique(X(X(:,4)>0,4)))' unique(X(X(:,4)>0,4)) X(xc,17) X(xc,1
8) xc];
    temp1 = group(group(:,1) == 1,:); % get all the 1 member groups
        if isempty(temp1) == 0
            temprest = group(group(:,1) > 1,:); % get all the multi-member groups
            if isempty(temprest) == 0
                X1 = repmat(temp1(:,3),1,size(temprest,1));
                Y1 = repmat(temp1(:,4),1,size(temprest,1));
                X2 = repmat(temprest(:,3)',size(temp1,1),1);
                Y2 = repmat(temprest(:,4)',size(temp1,1),1);
                dist = sqrt((X1-X2).^2 + (Y1-Y2).^2); % get distance between all singles (ver
tical) and multiples (horizontal)
                [~,mindistid] = min(dist,[],2);
                X(temp1(:,5),[4 17 18]) = temprest(mindistid,2:4); % change the group id & ho
me range centroid for the singles
            end
        end
    end
```

SAMPLING

```
         infid = 1:size(X,1);%find(X(:,17) > sampspace(2,1) & X(:,17) <= sampspace(2,2)); % i
ds for all individuals in infected cells
         %uninfid = find(X(:,17) > sampspace(1,1) & X(:,17) <= sampspace(1,2));
         SEIR(i,1:4) = [sum(X(infid,25) > 0) sum(X(infid,26) > 0) sum(X(infid,26) == 0 & X(in
fid,29) == 0) sum(X(infid,26) > 0 | X(infid,29) > 0)];%...live, dead
             %sum(X(uninfid,26) == 0 & X(uninfid,29) == 0) sum(X(uninfid,26) > 0 | X(uninfid,
29) > 0)]; %live inf zone, dead inf zone, live buff zone, dead buff zone

% Record pop size available for sampling: live, dead diseased, dead negative buffer and infec
ted zones
  if sum(ss(i,:)) > 0
    samp(i,:) = [round(sampprob(i,1).*SEIR(i,3)) round(sampprob(i,2).*SEIR(i,4))];% round(sam
pprob(i,3).*SEIR(i,5)) round(sampprob(i,4).*SEIR(i,6))]; % get scaled sample size for infecte
d zone
```

```
    [outid,distid] = sampling3(X,samp(i,:),bordercoor);%); % % live inf, dead inf, live buff,
 dead buff
    death(i,4) = length(nonzeros(outid(:,1)));%+length(nonzeros(outid(:,3))); % number sample
d by hunters
    % record numbers sampled for infec zone (live = outid(:,1), dead = outid(:,2))
    for kk = 1:2
        temp = nonzeros(outid(:,kk)); % get the vector of ids to be sampled - infected zone
        %temp2 = nonzeros(outid(:,kk+2)); % get the vector of ids to be sampled - buffer zone
        if isempty(temp) == 0 % if there are some, determine the number of positives and nega
tives
            surv(i,kk) = length(find(sum(X(temp,25:26),2) > 0)); %positives (live, dead) - in
fected zone
            surv(i,kk+2) = length(find(sum(X(temp,25:26),2) == 0)); %negatives (live,dead) -
infected zone
            %surv(i,kk+6) = length(find(sum(X(temp2,25:26),2) > 0)); %positives (live, dead)
- buffer zone
            %surv(i,kk+8) = length(find(sum(X(temp2,25:26),2) == 0)); %negatives (live,dead)
- buffer zone
            %disp([i surv(i,1) surv(i,3)])
        end
    end
    %surv: # pos live, # pos dead, # neg live, # neg dead, max dist live,max dist dead
    surv(i,5:6) = distid(1,:); % max distances for infectious samples
    surv(i,7:8) = distid(2,:); % min distances for infectious samples
    all = unique(nonzeros(outid)); %get the unique id's of sampled individuals to remove perm
anently (include buffer zone here)
    X(all,:) = []; %remove sampled individuals permanently

  end


    %%%%%%%%%%%%%%%%%%%%%%%%%%%%%%%%%%%%%%%%%%%%%%%%%%%%%%%%%%%%%%%%%%%%%%%
```

NEW CONCEPTIONS - Density-dependent conception %%

```
    pops = histc(X(X(:,26)==0 & X(:,29)==0,27),net_list(:,1)); % get abundance of live indivi
duals per grid cell
    dif = X(:,28)-pops(X(:,27));
    id = find(X(:,12) == 1 & X(:,13) == 0 & X(:,14) > min_P & X(:,26) == 0 & X(:,29) == 0 & d
if > 0);

    %Get all reproductive-age, non-pregnant females, that have been non-pregnant longer than
the min time
    % between giving birth and conceiving again (version for
    % non-density-dependent births)
    %id = find(X(:,12) == 1 & X(:,13) == 0 & X(:,14) > min_P & X(:,26) == 0 & X(:,29) == 0);
% only allow if cell is not overpopulated
    % Weight successful conception by those who haven't in a while
    if isempty(id) == 0 && max(id) <= size(X,1)
        %disp(size(id))
        mat = [sort(rand(length(id),1),'ascend') sortrows([id X(id,14)],-2)]; %rand number, i
d, time since last conception
        con_id = mat(c_time(i) > mat(:,1),2); %get id's for those that conceive
        if isempty(con_id) == 0 % if there are new conceptions...
```

```
            X(con_id,[10 11 13 14]) = [L_size.*ones(length(con_id),1) gest.*ones(length(con_i
d),1) ones(length(con_id),1) zeros(length(con_id),1)]; % set litter size, gestation, preg clo
ck, post-natal clock
            %store(i,12) = length(con_id); % store the number of conceptions that happen toda
y
        end
    end
    %%%%%%%%%%%%%%%%%%%%%%%%%%%%%%%%%%%%%%%%%%%%%%%%%%%%%%%%%%%%%%%%%%%%%%
```

NEW BIRTHS %%%

```
    id = find(X(:,13) > X(:,11) & X(:,26) == 0); % get the alive, pregnant females that reach
ed their target gestation time
    if isempty(id) == 0 % if there are some births, get each litter and initialize each indiv
idual in it
        %newb = 0;
        for j = 1:length(id) % for each female's litter
           Y = zeros(X(id(j),10),size(X,2)); % make a matrix for the new individuals from mot
her id(j) based on the litter size
           Y(:,23) = 1; %make everyone susceptible
           Y(:,[27 28]) = repmat(X(id(j),[27 28]),size(Y,1),1); % assign the mother's habitat
 info

           ff = round(X(id(j),10)*0.5); mm = X(id(j),10)-ff; % determine the number of each s
ex in the litter
           Y(ff+1:ff+mm,3) = 1; % label the males
           Y(1:ff,[9 14]) = [L_age.*ones(ff,1) (min_P+1).*ones(ff,1)]; % minimum age at first
 reproduction, set initial post-natal clock to above P_min
           Y(:,[1 2 6 8]) = [max(X(:,1))+(1:size(Y,1))' ones(mm+ff,1) randsample(D_age,mm+ff,
1) round(randsample(long,size(Y,1),1))]; % add unique id, age, dispersal age, death age
           Y(:,[4 15 16 17 18]) = repmat(X(id(j),[4 17 18 17 18]),size(Y,1),1); % put everybo
dy in their mother's group and current HR
           X = [X; Y]; % add the newborns to the main population
           %newb = newb+size(Y,1); % store the new births
           % Keep track of some reproductive stats
%             X(id,20) = X(id,20)+1; %add 1 to litters
%             X(id,21) = X(id,21)+size(Y,1); %add offspring to litters
        end
        %store(i,13) = newb; % store the new births

        % Reset the pregnancy clocks, gestation time and litter size for females that gave bi
rth
        X(id,[10 11 13]) = 0;
        X(id,14) = 1; % start their post-natal clock
    end
    end % end if endi
    %%%%%%%%%%%%%%%%%%%%%%%%%%%%%%
    %set up some checks so that we don't continue if the run is looking
    %unrealistic (ONLY USE DURING ABC)
%       if SEIR(i,5) > Ecut % check if there are more than 100 newE in a day (unrealistic for
 our system)
%          endi = [endi;i];% make the loop stop if any of these situations occur
%       end %end if check > 0
%       if nansum(nansum(surv(1:i,1:2),2)) > Ocut % check if there are more than Ocut cases s
```

```
o far
%         endi = [endi;i];% make the loop stop if any of these situations occur
%       end %end if check > 0
%           if i > 180 && checkvec(i) == 1 %this will stop it if there are no cases for 3 mon
ths
%                check1 = nansum(SEIR(i-90:i,5));
%           end
%        if check1 == 0
%            endi = [endi;i];
%        end
%      if size(X,1) < popthreshold % this will stop it if the abundance is below a threshold
%         endi = [endi;i];
%      end % end is abundance > popthreshold
end % end for i

%if size(X,1) > popthreshold
```

SUMMARIZE THE OUTPUT

```
    cases = Retime(timeseries(:,1),cumdate,'sum');
    proptrans = Retime(timeseries(:,3),cumdate,'mean');
    abun = Retime(timeseries(:,2),cumdate,'point');
    truedist = Retime(timeseries(:,4),cumdate,'max');
    % MAX AND MIN DISTANCE OF POSITIVE SAMPLES (both buffer and infected zones)
    dist = [surv(:,5:6) surv(:,7:8)]; id = find(sum(surv(:,1:2),2) == 0); dist(id,:) = NaN;
    maxdistsamp = Retime(nanmax(dist(:,1:2),[],2),cumdate,'max');
    mindistsamp = Retime(nanmin(dist(:,3:4),[],2),cumdate,'min');

    % PREDICTED POSITIVE AND NEGATIVE CASES IN SAMPLES
    PosLiveInf = Retime(surv(:,1),cumdate,'sum'); % positive samples live
    PosDeadInf = Retime(surv(:,2),cumdate,'sum'); %positive samples dead
    %disp([PosLiveInf+PosDeadInf maxdistsamp])
    %disp([sum(PosLiveInf+PosDeadInf) nansum(SEIR(:,5)) max(SEIR(:,5)) size(X,1) min(endi)])
    NegLiveInf  = Retime(surv(:,3),cumdate,'sum'); %live neg
    NegDeadInf  = Retime(surv(:,4),cumdate,'sum'); %dead neg
%     PosLiveBuf = Retime(surv(:,7),cumdate,'sum'); % positive samples live
%     PosDeadBuf = Retime(surv(:,8),cumdate,'sum'); %positive samples dead
%     NegLiveBuf  = Retime(surv(:,9),cumdate,'sum'); %live neg
%     NegDeadBuf  = Retime(surv(:,10),cumdate,'sum'); %dead neg

%     % PREVALENCE FROM INFECTED ZONE ONLY
    prevalivesamp = PosLiveInf./(PosLiveInf+NegLiveInf); %LIVE
    prevdeadsamp = PosDeadInf./(PosDeadInf+NegDeadInf); %DEAD
    prevbothsamp = (PosLiveInf+PosDeadInf)./(PosLiveInf+PosDeadInf+NegLiveInf+NegDeadInf); %B
OTH

    % truth: dist max; dist min; prev in inf zone: both, live, dead; pos inf zone live,
            %dead; pos buff live dead; neg inf zone live,dead; neg buff live dead;
    % calculate metrics
% Monthly scale
    % Distance metrics, both zones
    x = [truth(:,1) maxdistsamp]; x(find(isnan(x)==1)) = 0;
    TAEdistmax = nansum(abs(diff(x,[],2)));
    x = [truth(:,2) mindistsamp]; x(find(isnan(x)==1)) = 0;
    TAEdistmin = nansum(abs(diff(x,[],2)));
    % Prevalence in the infected zone
```

```
    TAEprevboth = nansum(abs(truth(:,3)-prevbothsamp));
    TAEprevlive = nansum(abs(truth(:,4)-prevalivesamp));
    TAEprevdead = nansum(abs(truth(:,5)-prevdeadsamp));
    % Positives and negatives in both zones
    TAEposlive = nansum(abs(sum(truth(:,6),2)-(PosLiveInf))); % both zones
    TAEposdead = nansum(abs(sum(truth(:,7),2)-(PosDeadInf))); % both zones
    TAEneglive = nansum(abs(sum(truth(:,10),2)-(NegLiveInf)));
    TAEnegdead = nansum(abs(sum(truth(:,11),2)-(NegDeadInf)));
    TAEposboth = nansum(abs(sum(truth(:,6:7),2)-(PosLiveInf+PosDeadInf)));
% All time
    Tcases = abs(sum(PosLiveInf+PosDeadInf)-nansum(nansum(truth(:,6:7),2))); % predicted - ob
served
    Maxsamp = abs(max(0,max(maxdistsamp))-max(truth(:,1))); % predicted - observed

    timeseries = [maxdistsamp, mindistsamp, PosLiveInf, PosDeadInf, NegLiveInf, NegDeadInf, p
revalivesamp, prevdeadsamp, ...
        prevbothsamp, cases, proptrans, abun, truedist];

    met = [TAEdistmax,TAEdistmin,TAEprevboth,TAEprevlive,TAEprevdead,TAEposlive,TAEposdead,TA
Eposboth,TAEneglive,TAEnegdead,...
        Tcases,Maxsamp,min(endi),parmsin];

% calculate transmission matrix
%              TNET = zeros(length(uindID(:,1)),length(uindID(:,1)));
%              for tt = 1:time
%                  if isempty(nonzeros(cnet(:,tt,1))) == 0
%                      temp = [nonzeros(cnet(:,tt,1)) nonzeros(cnet(:,tt,2))];
%                      for kk = 1:size(temp,1)
%                          id1 = find(uindID(:,1) == temp(kk,1)); id2 = find(uindID(:,1) == te
mp(kk,2));
%                          TNET(id1,id2) = TNET(id1,id2) + 1;
%                      end
%                  end
%              end
%              id = find(TNET>0); TNET2 = TNET; TNET2(id) = 1; deg = sum(TNET2,2);
%              deg = [deg; nan(ndeg-length(deg),1)];
            deg = [];
            Re = [uindID(:,2); nan(ndeg-length(uindID(:,2)),1)];

end
```

```
Not enough input arguments.

Error in main_predict_DenDepRepro2 (line 67)
ss = [samples(:,4) sum(samples(:,5:6),2)]; %inf zone
```

Published with MATLAB® R2019b
