# Supplementary material for: Ecological drivers of African swine fever virus persistence in wild boar populations: Insight for control
Source: Ecol Evol. 2020 Feb 18;10(6):2846–59. doi: 10.1002/ece3.6100 (PMC7083705; doi:10.1002/ece3.6100)
Supplement: Supplementary file 4 [file ECE3-10-2846-s004.pdf]

```
function [outid,distid] = sampling3(X,samp,bordercoor)
%surveillance sampling of population
% save('SamplingVector','samples');
% sampling compartments: hunted infected, carcass infected
% X: matrix of individuals
% samp: number of samples collected form each compartment (above)
% outid: id's of pigs to sample from each category (once sampled, remove)
% distid: X coordinate of each id sampled

outid = zeros(12000,length(samp)); % matrix to store individual id's for sampling

for j = 1:length(samp)
    if j == 1
        id = find(sum(X(:,[26 29]),2) == 0 & X(:,2) >= 180);
    elseif j == 2
        id = find(sum(X(:,[26 29]),2) > 0 & X(:,2) >= 90);

    end
        sampsize = samp(j);
        if length(id) >= sampsize && sampsize > 0
            outid(1:sampsize,j) = randsample(id,sampsize);
        elseif length(id) >= 1 && sampsize > 0 && length(id) < sampsize
            outid(1:length(id),j) = id; % if there aren't enough, sample all
        end % if there are none to sample, don't do anything
end

% For each set of id's to be sampled, get the max dist from the border
distid = zeros(2,length(samp));
for j = 1:size(distid,2)
    temp = X(nonzeros(outid(:,j)),[17 25 26 18]);
    temp2 = sum(temp(:,2:3),2) > 0;
    if sum(temp2) > 0 % if there is at least one infectious sample
        id = find(temp2 > 0); % get ids of infectious individuals
        % get distance matrix of cases from border coordinates and take minimum (to
            % get distance of cases to border)
        mat = sqrt((temp(id,1)-repmat(bordercoor(:,1)',length(id),1)).^2 + (temp(id,4)-repmat
(bordercoor(:,2)',length(id),1)).^2);
        dd = min(mat,[],2); % distance from the border for each case
        distid(1,j) = max(dd); % maximum distance from border
        distid(2,j) = min(dd); % min distance from border
    else
        distid(1:2,j) = nan;
    end
end
end
```

```
Not enough input arguments.

Error in sampling3 (line 10)
outid = zeros(12000,length(samp)); % matrix to store individual id's for sampling
```

Published with MATLAB® R2019b
