# Supplementary material for: Ecological drivers of African swine fever virus persistence in wild boar populations: Insight for control
Source: Ecol Evol. 2020 Feb 18;10(6):2846–59. doi: 10.1002/ece3.6100 (PMC7083705; doi:10.1002/ece3.6100)
Supplement: Supplementary file 5 [file ECE3-10-2846-s005.pdf]

```
function [Eid,Eidc,probC,critC,probD,critD] = vectorize3(out,Iid,Sid,Cid,TPGC_d,TPc,movement)
%VECTORIZE THE TRANSMISSION CODE
%   TPc = TPGC_c*carcass
% Between is the contact rate for individuals in different groups at
% different distances
% Within is the contact rate for individuals in the same group
% Space is the PDF for daily movements
% style is the method for doing contact/transmission
% TPGC_d/TP_c are the transmission probabilities given contact
% movement defines the parameters or PDF of daily movement

% CHOOSE A METHOD FOR IMPLEMENTING CONTACT
% 1) LOCAL NEIGHBORHOOD: contact is only within a fixed distance, all
% individuals have the same fixed distance
% 2) DISTANCE KERNEL: similar to local neoghborhood but contact probability
% decreases with distance in the neighborhood
% 5) VARIABLE LOCAL NEIGHBORHOOD: local neighborhood size changes based on
% daily movement distribution, equal between-group contact prob within the local
% neighborhood, higher contact rates within groups
% Note method 5 used for all sims in "Ecological drivers of African swine
% fever virus persistence in wild boar populations: insight for control "

probD = rand(length(Sid),length(Iid)); %get random number that will determine whether contact
 leads to transmission for direct contact
probC = rand(length(Sid),length(Cid)); % get random number that will determine whether contac
t leads to transmission for carcass contact

% transmission criteria for spatial contact
critD = zeros(length(Sid),length(Iid)); % make matrix with transmission criteria that account
 for contact heterogeneity
critC = zeros(length(Sid),length(Cid)); % make matrix with transmission criteria that account
 for contact heterogeneity

ss = movement; %contact style, fixed radius, decay, within for direct, within for carcass
style = ss(1);
switch style
    case 1 % contact everyone on the fixed distance neighborhood (no distinction between same
 group or not):
         % ss = 1 X 2; case, fixed radius
        if isempty(Iid) == 0
            critD = ones(length(Sid),length(Iid)).*TPGC_d; % give everyone in the radius the
same transmission probability
            distIidX = repmat(out(Iid,17)',length(Sid),1); % matrix with X coordinates of Iid
s
            distIidY = repmat(out(Iid,18)',length(Sid),1); % matrix with Y coordinates of Iid
s
            distSidX = repmat(out(Sid,17),1,length(Iid)); % matrix with X coordinates of Sids
            distSidY = repmat(out(Sid,18),1,length(Iid)); % matrix with Y coordinates of Sids
            dist = sqrt((distSidX-distIidX).^2 + (distSidY-distIidY).^2); % get distance betw
en S and each I
            critD(dist>ss(2)) = 0; % give no probability of transmission if home range centro
ids are not within fixed distance
        end
        if isempty(Cid) == 0
            critC = ones(length(Sid),length(Cid)).*TPc;
```

```
            distCidX = repmat(out(Cid,17)',length(Sid),1); % matrix with X coordinates of Iid
s
            distCidY = repmat(out(Cid,18)',length(Sid),1); % matrix with Y coordinates of Iid
s
            distSidX = repmat(out(Sid,17),1,length(Cid)); % matrix with X coordinates of Sids
            distSidY = repmat(out(Sid,18),1,length(Cid)); % matrix with Y coordinates of Sids
            dist = sqrt((distSidX-distCidX).^2 + (distSidY-distCidY).^2); % get distance betw
en S and each I
            critC(dist>ss(2)) = 0; % give no probability of transmission if home range centro
ids are not within fixed distance
        end
    case 2 % probability of contact decays with distance (don't distinguish within or between
 group): just TP*exp(-rd)
      % ss = 1 x 2; case, fixed decay rate
        if isempty(Iid) == 0
            distIidX = repmat(out(Iid,17)',length(Sid),1); % matrix with X coordinates of Iid
s
            distIidY = repmat(out(Iid,18)',length(Sid),1); % matrix with Y coordinates of Iid
s
            distSidX = repmat(out(Sid,17),1,length(Iid)); % matrix with X coordinates of Sids
            distSidY = repmat(out(Sid,18),1,length(Iid)); % matrix with Y coordinates of Sids
            dist = sqrt((distSidX-distIidX).^2 + (distSidY-distIidY).^2); % get distance betw
en S and each I
            critD = TPGC_d.*exp(-ss(3).*dist); % probability of contact decays with distance
        end
        if isempty(Cid) == 0
            distCidX = repmat(out(Cid,17)',length(Sid),1); % matrix with X coordinates of Iid
s
            distCidY = repmat(out(Cid,18)',length(Sid),1); % matrix with Y coordinates of Iid
s
            distSidX = repmat(out(Sid,17),1,length(Cid)); % matrix with X coordinates of Sids
            distSidY = repmat(out(Sid,18),1,length(Cid)); % matrix with Y coordinates of Sids
            dist = sqrt((distSidX-distCidX).^2 + (distSidY-distCidY).^2); % get distance betw
en S and each I
            critC = TPc.*exp(-ss(3).*dist); % probability of contact decays with distance
        end


      case 5 % probability of contact decays with distance (don't distinguish within or betwe
en group): just TP*exp(-rd) and group structure
      % ss = 1 x 4; case, fixed decay rate
        if isempty(Iid) == 0
            distIidX = repmat(out(Iid,17)',length(Sid),1); % matrix with X coordinates of Iid
s
            distIidY = repmat(out(Iid,18)',length(Sid),1); % matrix with Y coordinates of Iid
s
            distSidX = repmat(out(Sid,17),1,length(Iid)); % matrix with X coordinates of Sids
            distSidY = repmat(out(Sid,18),1,length(Iid)); % matrix with Y coordinates of Sids
            dist = sqrt((distSidX-distIidX).^2 + (distSidY-distIidY).^2); % get distance betw
en S and each I
            critD = TPGC_d.*exp(-ss(3).*dist); % probability of contact decays with distance
for between-group transmission
            groupSid = repmat(out(Sid,4),1,length(Iid));
            groupIid = repmat((out(Iid,4))',length(Sid),1); % make a matrix of sounder id's t
o label within or between
            % Within group tranmission
            witID = groupIid == groupSid; % matrix delineating within-sounder contacts
```

```
            critD(witID == 1) = ss(4); % assign within-group contact probability
        end
        if isempty(Cid) == 0
            distCidX = repmat(out(Cid,17)',length(Sid),1); % matrix with X coordinates of Iid
s
            distCidY = repmat(out(Cid,18)',length(Sid),1); % matrix with Y coordinates of Iid
s
            distSidX = repmat(out(Sid,17),1,length(Cid)); % matrix with X coordinates of Sids
            distSidY = repmat(out(Sid,18),1,length(Cid)); % matrix with Y coordinates of Sids
            dist = sqrt((distSidX-distCidX).^2 + (distSidY-distCidY).^2); % get distance betw
en S and each I
            critC = TPc.*exp(-ss(3).*dist); % probability of contact decays with distance
            groupSidC = repmat(out(Sid,4),1,length(Cid));
            groupCid = repmat((out(Cid,4))',length(Sid),1); % make a matrix of sounder id's t
o label within or between
            % Within group tranmission
            witID = groupCid == groupSidC; % matrix delineating within-sounder contacts
            critC(witID == 1) = ss(5); % assign within-group contact probability
        end


end
%disp([size(probD) size(critD)])
Eid = unique(find(sum(probD<critD,2) == 1)); %these are the S that will become E by direct co
ntact (they had at least one successful transmission)
Eidc = unique(find(sum(probC<critC,2) == 1)); %these are the S that will become E by carcass
contact (they had at least one successful transmission)

%Eid,Eidc,probC,critC,probD,critD: these are outputs so don't clear
clear vec dist witID betID dmS ss groupSid groupCid distCidX distCidY distSidX distSidY distI
idX distIidY;

end
```

```
Not enough input arguments.

Error in vectorize3 (line 23)
probD = rand(length(Sid),length(Iid)); %get random number that will determine whether contact
 leads to transmission for direct contact
```

Published with MATLAB® R2019b
