# Supplementary material for: Ecological drivers of African swine fever virus persistence in wild boar populations: Insight for control
Source: Ecol Evol. 2020 Feb 18;10(6):2846–59. doi: 10.1002/ece3.6100 (PMC7083705; doi:10.1002/ece3.6100)
Supplement: Supplementary file 6 [file ECE3-10-2846-s006.pdf]

```
function [DATA,Sid,Eid,Eidc,pairs] = dis3(X,TPGC_d,TPc,movement,dayi)

% contact & transmission (only if there are I's and S's)
    Iid = find(X(:,25) > 0); % get infectious individuals
    Sid = find(X(:,23) == 1); % get susceptible individuals
    Cid = find(X(:,26) > 0); % get infectious carcasses

    [Eid,Eidc,probC,critC,probD,critD] = vectorize3(X,Iid,Sid,Cid,TPGC_d,TPc,movement);

% Collect infection data similar to actual data
% For each new case: Day, ID, Age, Sex, Group ID, X, Y, Grid cell ID, Type of transmission
DATA = [dayi.*ones(length(Eid),1) X(Sid(Eid),[1 2:4 17 18 27]) ones(length(Eid),1);...
    dayi.*ones(length(Eidc),1) X(Sid(Eidc),[1 2:4 17 18 27]) 2.*ones(length(Eidc),1)];

% Determine transmission pairs for network analysis
Is = [Iid; Cid]; mat = [probD probC] < [critD critC];
pairs = [];
for i = 1:length(Sid)
 id = find(mat(i,:) == 1);
   if isempty(id) == 0
     pairs = [pairs; X(Sid(i),1).*ones(length(id),1) X(Is(id),1)]; %ids of recipient and dono
r pairs
   end
end
clear Iid Cid;
end
```

```
Not enough input arguments.

Error in dis3 (line 4)
    Iid = find(X(:,25) > 0); % get infectious individuals
```

Published with MATLAB® R2019b
